# Editorial: Functional Characterization and Pharmaceutical Targets in Common and Rare CFTR Dysfunctions

**DOI:** 10.3389/fphys.2021.830285

**Published:** 2022-01-24

**Authors:** Elena K. Schneider-Futschik, Viola H. Lobert, John W. Wilson

**Affiliations:** ^1^Cystic Fibrosis Pharmacology Laboratory, Department of Biochemistry & Pharmacology, Melbourne University, Melbourne, VIC, Australia; ^2^Department of Nursing, Health and Laboratory Science, Østfold University College, Fredrikstad, Norway; ^3^Department of Mechanical, Electronic and Chemical Engineering, OsloMet, Oslo, Norway; ^4^Department of Medicine, The Alfred Hospital, Monash University, Melbourne, VIC, Australia; ^5^Cystic Fibrosis Service, The Alfred Hospital, Melbourne, VIC, Australia

**Keywords:** CFTR (cystic fibrosis transmembrane conductance regulator), CFTR (cystic fibrosis transmembrane regulator), cystic fibrosis, lung, biomarker

## Introduction

Cystic fibrosis (CF) is a life-limiting multisystem disease caused by a dysfunction in the cystic fibrosis transmembrane conductance regulator protein (CFTR). CF is associated with a myriad of respiratory complications, notably, an increased susceptibility to lung infections and inflammation (Schneider et al., 2017). Sustained and progressive inflammatory insults lead to continuous airway damage and remodeling, resulting in compromised lung function.

Since the discovery of the CFTR gene and the resulting understanding of cellular and molecular defects of CF-causing mutations, new perspectives for the development of novel therapies have changed the field of CF (Kerem et al., 1989). Currently, four new drug combinations that target the root cause of CF have been brought to market: ivacaftor monotherapy and ivacaftor combinations with lumacaftor, tezacaftor or tezacaftor-elexacaftor (Ghelani and Schneider-Futschik, 2020). Together with supplementary therapies and early diagnosis, the quality of life and life expectancy of patients has significantly increased (Lopes-Pacheco, 2016).

This Research Topic gathers a collection of 7 original and review articles that provide novel information regarding the “Functional Characterization and Pharmaceutical Targets in Common and Rare CFTR Dysfunctions” at basic, translational, and clinical levels. The articles have been divided in three main chapters.

The first chapter gives some insights on differences in corrector efficacy/corrector sensitivity due to protein misfolding and nonsense-mediated decay. Nonsense mutations are caused by an insertion of a premature stop codon in the CFTR transcript which affects ~11% of CF patients worldwide and result in a severe disease phenotype. Sharma et al. developed and characterized the first homozygous CF rat model that bears the CFTR G542X nonsense mutation in the endogenous locus using CRISPR/Cas9 gene editing. The G542X rat model displays severe CF manifestations and developmental defects (resulting of low CFTR mRNA levels). Even though the authors reported functional restoration of defective CFTR using therapeutic agents, therapeutic efficacy was not observed. The authors suggest that the G542X rat model provides a novel tool for the identification and *in vivo* validation of potential therapies. Peters et al. focused on whether the response of rare CFTR folding mutations to correctors can be improved. The research group has previously identified that the effects of SUMO-1-regulation can increase the impact of correctors on F508del CFTR (Gong et al., 2019). However, it was unknown whether this extended to other rare CFTR folding mutations. Small modifier-like modifier, SUMO, is a post-translational modification that regulates the degradation of cytosolic proteins. F508del-CFTR has been previously observed to be degraded via the SUMO-dependent pathway (Ahner et al., 2013). In this study, 17 CFTR mutants were tested in a bronchial epithelial cell line with or without the over-expression of the SUMO E3 ligase PIAS4 and the corrector C18. Several mutants were found to have an amplified response to correctors when PIAS4 was overexpressed, suggesting that modifying the SUMOylation pathway may be a useful approach to be targeted by small molecules.

The second chapter is dedicated to new approaches that play a key role in the regulation of CFTR. Veltman et al. examined the impact of CFTR deficiency on lipid metabolism and pro-inflammatory signaling. They report a prominent imbalance in fatty acid and ceramide metabolism associated with chronic oxidative stress using different cell-based models including mouse lung and differentiated human or pig epithelial cell. CFTR deficiency is likely to cause oxidative stress in CF airway epithelium, affecting multiple bioactive lipid metabolic pathways, which likely play a role in CF lung disease progression (Veltman et al.). Cui et al. showed that cholesterol, an important membrane lipid component, modulates the function of CFTR (Kopp et al., 2020). The authors utilized methyl-β-cyclodextrin to reduce accessible and total cholesterol in the plasma membrane of polarized fischer rat thyroid and human bronchial epithelial cells to subsequently activate these cells with forskolin. They showed that changes in the plasma membrane cholesterol levels changes the function of the CFTR channel and consequently, affects sensitivity to clinical therapeutics such as ivacaftor.

A review by Della Sala et al. nicely summarizes the role of protein Kinase A-mediated phosphorylation in CFTR channel activity and provides insights into the different factors that modulate its function. The CFTR channel is an anion channel found on apical membranes of epithelial cells, and while PKA has been known to be important for CFTR activation since the 1990s, some novel aspects of this regulation have emerged (Egan et al., 1992). The mechanism of action of many modulators has been attributed to restoring CFTR expression, folding, and activity of defective CFTR variants. Importantly, PKA-mediated phosphorylation of CFTR regulates several aspects of CFTR trafficking and activity, but how modulators affect PKA-mediated CFTR mutant phosphorylation has remained unclear. The authors present some recent papers that suggest that the modulator action may in fact be dependent on the phosphorylation of the channel. Targeting the PKA-mediated phosphorylation of CFTR may therefore be a promising therapeutic strategy in order to improve modulator efficacy.

Nietert et al. provides readers with a database, CandActCFTR, that lists candidate therapeutics that will activate CFTR in a synergistic fashion based on published data. This database is not restricted to a particular CFTR mutation type or to a particular dataset, but merges publicly available information. While this database focuses on CFTR, the authors hope that this initiative will inspire others to start similar approaches for other purposes/diseases. This database aims to fill a current gap, since there is currently no comprehensive database to find chemical compounds.

The final chapter looks at biomarkers for best diagnosis/prognosis for CF. Most cases of CF are diagnosed early in life, often via newborn screenings or clinical manifestations of symptoms. However, cases with atypical CF such as normal or borderline sweat tests or without the identification of known CFTR mutations require further confirmation or exclusion of diagnosis. Silva et al. analyzed CFTR-mediated chloride secretion in rectal biopsies from 143 patients. They found that the IBMX (I)/Forskolin (F)/Carbachol (C)-stimulated equivalent short-circuit current, is the most sensitive, reproducible, and robust predictive biomarker for CF diagnosis/prognosis effectively differentiating between classical, atypical CF and non-CF groups.

## Conclusion

In conclusion, this Research Topic collates current knowledge on the functional characterization and pharmaceutical targets in common and rare CFTR mutations. These range from novel insights into the basic function of CFTR to translational and clinical biomarker studies involving diagnosis and prognosis of patients with CF. With the breadth of work currently being conducted in the field of CFTR characterisations, the horizon looks optimistic for several approaches to translate into real-life patient applications.

## Author Contributions

ES-F, VL, and JW wrote and reviewed the Editorial. All authors contributed to the article and approved the submitted version.

## Funding

ES-F was supported by the NHMRC (Grant ID: APP1157287) and Cystic Fibrosis Australia.

## Conflict of Interest

JW had received clinical trial and consultancy funding from Vertex. The remaining authors declare that the research was conducted in the absence of any commercial or financial relationships that could be construed as a potential conflict of interest.

## Publisher's Note

All claims expressed in this article are solely those of the authors and do not necessarily represent those of their affiliated organizations, or those of the publisher, the editors and the reviewers. Any product that may be evaluated in this article, or claim that may be made by its manufacturer, is not guaranteed or endorsed by the publisher.
